# Supplementary material for: Causal Relationship between Aging and Anorexia Nervosa: A White-Matter-Microstructure-Mediated Mendelian Randomization Analysis
Source: Biomedicines. 2024 Aug 16;12(8):1874. doi: 10.3390/biomedicines12081874 (PMC11351342; doi:10.3390/biomedicines12081874)
Supplement: Supplementary file 1 [file biomedicines-12-01874-s001.zip › Supplementary Figure/Figure S4/Singe SNP plot of MR analysis for TL to ubm-b-1884.pdf]

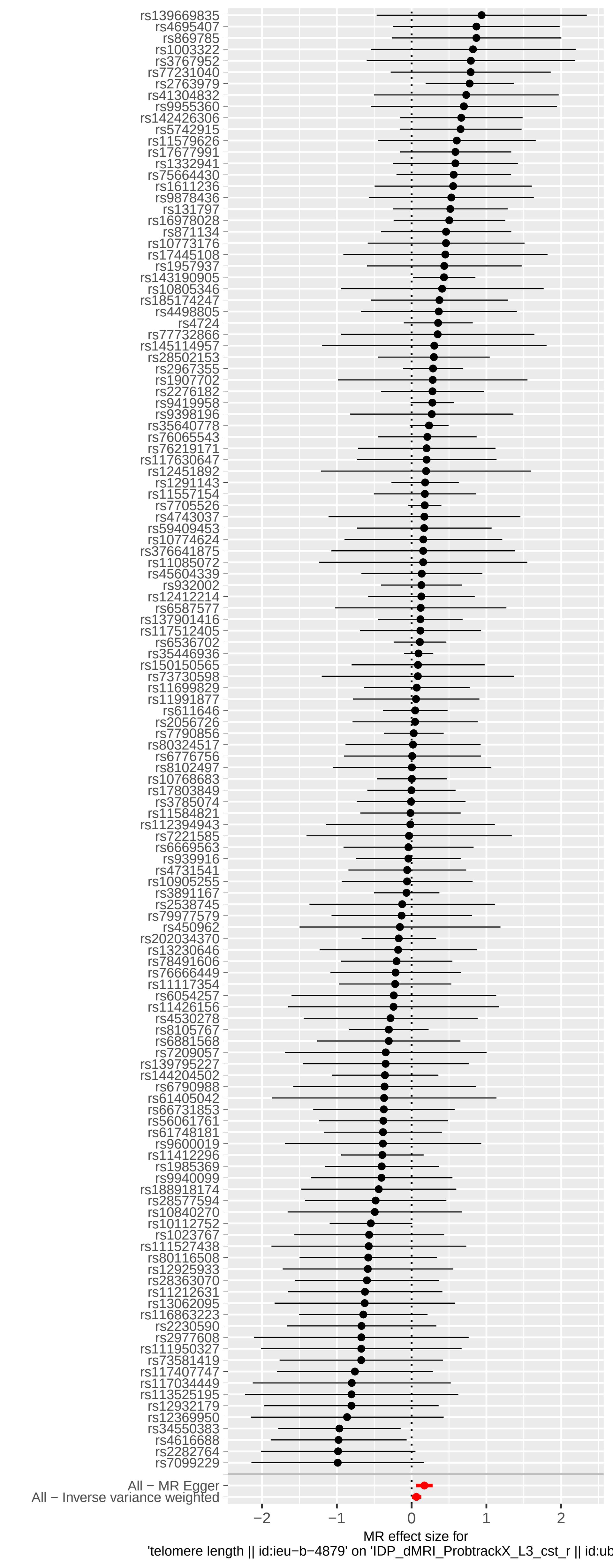

rs139669835
rs4695407
rs869785
rs1003322
rs3767952
rs77231040
rs2763979
rs41304832
rs9955360
rs142426306
rs5742915
rs11579626
rs17677991
rs1332941
rs75664430
rs1611236
rs9878436
rs131797
rs16978028
rs871134
rs10773176
rs17445108
rs1957937
rs143190905
rs10805346
rs185174247
rs4498805
rs4724
rs77732866
rs145114957
rs28502153
rs2967355
rs1907702
rs2276182
rs9419958
rs9398196
rs35640778
rs76065543
rs76219171
rs117630647
rs12451892
rs1291143
rs11557154
rs7705526
rs4743037
rs59409453
rs10774624
rs376641875
rs11085072
rs45604339
rs932002
rs12412214
rs6587577
rs137901416
rs117512405
rs6536702
rs35446936
rs150150565
rs73730598
rs11699829
rs11991877
rs611646
rs2056726
rs7790856
rs80324517
rs6776756
rs8102497
rs10768683
rs17803849
rs3785074
rs11584821
rs112394943
rs7221585
rs6669563
rs939916
rs4731541
rs10905255
rs3891167
rs2538745
rs79977579
rs450962
rs202034370
rs13230646
rs78491606
rs76666449
rs11117354
rs6054257
rs11426156
rs4530278
rs8105767
rs6881568
rs7209057
rs139795227
rs144204502
rs6790988
rs61405042
rs66731853
rs56061761
rs61748181
rs9600019
rs11412296
rs1985369
rs9940099
rs188918174
rs28577594
rs10840270
rs10112752
rs1023767
rs111527438
rs80116508
rs12925933
rs28363070
rs11212631
rs13062095
rs116863223
rs2230590
rs2977608
rs111950327
rs73581419
rs117407747
rs117034449
rs113525195
rs12932179
rs12369950
rs34550383
rs4616688
rs2282764
rs7099229
All − MR Egger
All − Inverse variance weighted
−2
−1
0
1
2
MR effect size for
'telomere length || id:ieu−b−4879' on 'IDP_dMRI_ProbtrackX_L3_cst_r || id:ub
